# Neural oscillations and event-related potentials reveal how semantic congruence drives long-term memory in both young and older humans

**DOI:** 10.1038/s41598-020-65872-7

**Published:** 2020-06-04

**Authors:** Pau A. Packard, Tineke K. Steiger, Lluís Fuentemilla, Nico Bunzeck

**Affiliations:** 1 Institute of Psychology I, University of Lübeck, 23562 Lübeck, Germany; 2 Cognition and Brain Plasticity Group, Bellvitge Biomedical Research Institute (IDIBELL), Hospitalet de Llobregat, Barcelona, Spain; 3 Department of Cognition, Development and Educational Psychology, University of Barcelona, Barcelona, Spain; 4 Institute of Neurosciences, University of Barcelona, Barcelona, Spain

**Keywords:** Long-term memory, Human behaviour

## Abstract

Long-term memory can improve when incoming information is congruent with known semantic information. This so-called congruence effect has widely been shown in younger adults, but age-related changes and neural mechanisms remain unclear. Here, congruence improved recognition memory in younger and older adults (i.e. congruence effect), with only weak evidence for age-related decline in one behavioral study. In an EEG study, however, no significant behavioral differences in the congruence effect could be observed between age-groups. In line with this observation, electroencephalography data show that, in both groups, congruence led to widespread differences in Event-Related Potentials (ERPs), starting at around 400 ms after stimulus onset, and theta, alpha and beta oscillations (4–20 Hz). Importantly, these congruence-related ERPs were associated to increases in memory performance for congruent items, in both age groups. Finally, the described ERPs and neural oscillations in the theta-alpha range (5–13 Hz) were less pronounced in the elderly despite a preserved congruence effect. Together, semantic congruence increases long-term memory across the lifespan, and, at the neural level, this could be linked to neural oscillations in the theta, alpha and beta range, as well as ERPs that were previously associated with semantic processing.

## Introduction

Long-term memory can be improved by presenting the information that has to be learned within a known context. In humans, this has often been demonstrated with a priori semantic information followed by congruent (vs. incongruent) material, and has, therefore, been labeled semantic congruence effect (or simply ‘congruence effect’)^1–8^. While most previous studies have focused on younger participants (i.e. 18–35 years), the potential age-related changes and associated neural mechanisms, in particular neural oscillations, remain unclear. While age-related impairments could be expected on the basis of well-described memory deficits in older adults, it is also clear that semantic memory (i.e. long-term memory for facts independent of time and date) is often preserved until old age^9^. Therefore, we investigated the semantic congruence effect in both young and older adults, as well as the underlying neural processes, including event-related potentials (ERPs) and time-frequency analyses, using EEG.

The processing of congruent semantic information may lead to better memories^10,11^. At the neural level, there is increasing support for a basic congruence dependent mechanism associated with the integration of memories into long-term knowledge structures or schemas^8,12–19^. According to this framework, the semantic congruence matching during encoding may be an initial step in such a general schema-dependent process of memory integration which entails interactions between the medial temporal lobe and the prefrontal cortex that favor an efficient retention and a faster consolidation of congruent events. Schema-related memory theories also nicely complement theories which emphasize the anticipatory, constructive nature of cognition and memory^20–22^. According to this view, incoming information is linked to representations in memory that pre-activate associations, thus forming predictions and selectively facilitating cognition. Therefore, general knowledge stored in neural networks plays an important role in guiding the selection of the inputs that are meaningful according to goals.

Post-stimulus ERPs during encoding reflect memory processes that are modulated by earlier input or the configuration of activity in memory^23,24^. In this regard, the frontal N400 (FN400), is often associated with familiarity processing^25^, but also with conceptual priming^26^. A similar component (in terms of temporal dynamics and topography), the N400, is typically associated with semantic memory processes^27,28^. The Late Positive Component (LPC), on the other hand, typically emerges from 400 to 800 ms, and has been shown to index recollective processing^29,30^. Moreover, item memory strength (confidence) correlates with ERP differences in the 300–500 ms time window (FN 400), whereas source memory strength correlates with the LPC^31^. Semantic congruence also modulates early encoding-related post-stimulus positive ERPs^5^. Finally, the term “Difference based on later Memory” (DM) was coined to define the increased positivity of ERPs elicited during the encoding of words that were later remembered compared to words that were later forgotten^32^.

Older adults exhibit several changes in ERPs, which provide insights into related cognition, and the neural processes underlying them. Importantly, age-related decreases in ERPs that are associated with semantic congruence appear to reflect an age-related deterioration in neural processes^33–35^. Moreover, the N400 was found to be lower in older than in younger adults, which might reflect changes in processing of meaning (i.e., world-knowledge) and semantic memory^27^. In addition, older adults have reduced DM ERPs within the first second after encoding^36,37^. Finally, in amnesic patients, the FN400, putatively related to familiarity-processing, is relatively spared compared to the LPC, generally associated to episodic memory^38^. And, in patients with Alzheimer’s disease and mild cognitive impairment, abnormalities in the late positive P600 appear at early stages, with changes in the P300 and N400 being more common at later stages^39,40^.

In terms of neural oscillations, the beta band (16–25 Hz) has been suggested to support the maintenance of events necessary for memory encoding^41^. In addition, alpha-beta (8–30 Hz) oscillations may underlie encoding, possibly reflecting an increase in information^42,43^, or controlled access to matching information in semantic memory^44^. In general terms, theta (4–8 Hz) and alpha (8–13 Hz) oscillations closely relate to memory performance^45^, and age-related changes lead to memory deficiencies^46^. Theta-alpha oscillations may also support the binding of information across large-scale networks including the prefrontal cortex and medial temporal lobe structures^47,48^. Finally, theta oscillations are involved in integrative encoding^49^, and may mediate the positive semantic congruence effect for episodic memories and explain age-related declines^1,50,51^

Here, we used electroencephalography (EEG) to investigate the temporal dynamics of the neural correlates associated with age-related differences in the semantic congruence effect. To this end, we implemented an adapted word list paradigm from a previous study^5^, in which a strong memory enhancement was found for congruent words, with a corresponding early appearing ERP during encoding. Participants were presented with a series of word pairs: the first word was a semantic category (e.g. furniture) designed to preactivate specific semantic memory networks. The second word was an item either congruent (e.g. chair) or incongruent (e.g. apple) with the previous category. Participants classified the congruence vs incongruence of the second word and their long-term recognition memory was tested in a separate subsequent phase. We expected a weaker congruence effect in older adults as well as specific and age-related effects in post-stimulus ERPs, theta, alpha and possibly beta oscillations.

## Materials and Methods

### Participants

Thirty young (ages 19–33 years, mean 23.87; SD 3.53, 14 males) and twenty-eight older participants (ages 50–79 years, mean 62.55; SD 7.02, 13 males) were recruited for the behavioral experiment (Experiment 1). Before the analysis of Experiment 1, one subject with a MOCA score of 20 was excluded. Subsequently, twenty-three young (ages 18–28 years, mean 20.95; SD 3.23, 11 males) and twenty-five older participants (ages 52–79 years, mean 63.21; SD 5.82, 11 males) were recruited for the EEG experiment (Experiment 2).

All participants were healthy, right-handed, had normal or corrected-to-normal vision (including color-vision) and reported no history of neurological or psychiatric disorders, or current medical problems (excluding blood pressure). All older participants scored a 22 or higher on the Montreal Cognitive Assessment version 7^52^. A cutoff value of 22 was chosen based on a study^53^ that recommends this value as an appropriate cut-off for mild cognitive impairment. There was no significant statistical difference in the mean MoCA scores between the older group in Experiment 1 (ranging from 22 to 29) and the older group in Experiment 2 (ranging from 22 to 30). Note that we also analyzed all behavioral data with a MoCA cutoff of 26 (i.e. excluding older participants with a MoCA of less than 26). It revealed very similar findings as compared to the initial analysis (see supplementary findings).

Participants were recruited through announcements in the local newspaper or the database of the University of Lübeck^54^. All participants signed a written informed consent and received monetary compensation. The study was approved by the local ethical committee of the University of Lübeck, Germany, and in accordance with the Declaration of Helsinki.

### Materials

Experimental stimuli consisted of 66 word lists from a previous study^5^ translated into German, selected from category norms^55–57^. Each list consisted of the 6 most typical instances (e.g., cow, pig, horse, chicken, sheep, and goat) of a natural/artificial category (e.g., farm animal). All of the 396 typical instances, semantically related to their respective semantic categories, were presented in separate encoding trials, each time preceded by a category (semantic cue). Additionally, semantically unrelated words were used as control words (new words) in the test phase.

### Behavioral procedures

**Figure 1:**
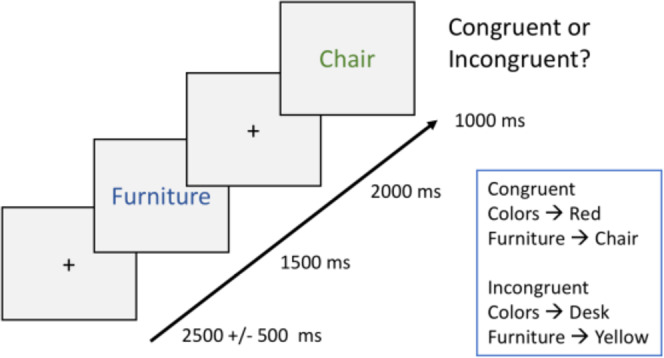
Encoding task. 288 trials were presented. Each trial began with the presentation of a fixation cross, then the presentation of a semantic category in blue font, another fixation cross, and finally a word item in green. Participants responded with a mouse click for each green word, they were instructed to identify, as fast as possible, whether the word belonged to the preceding category (congruent), or not (incongruent). The encoding phase included 288 trials in random order, with both conditions (congruent or incongruent, 144 trials each) randomly mixed. Typical examples of each condition are shown. Timings are shown in ms.

We used a modified version of a previous EEG experiment^5^ itself adapted from other paradigms^56,58,59^. Here, participants were first presented with a series of word pairs (i.e. study phase), this was followed by an informed recognition memory test (see Fig. 1). They observed the screen from a distance of ca. 50 cm on a display with a diagonal of 62 cm. Arial letter type, 36-letter size was used.

The study phase consisted of 396 separate word-encoding trials, presented mixed in random order. Each trial started with the appearance of a fixation cross on the screen for a random duration of 2000–3000 ms. Subsequently, a category name in blue appeared on a white background for 1500 ms. After the cue disappeared, a fixation cross appeared for 2000 ms. Participants were then sequentially shown the subsequent word in green for 1000 ms. In the congruent condition, the subsequent word belonged to the semantic category^2^, for example, ‘colors’ followed by ‘blue’; or ‘furniture’ followed by ‘desk’. In the incongruent condition, the category name did not correspond to the subsequent word, for example ‘planets’ followed by ‘cottage’; or ‘continents’ followed by ‘oxygen’. While the second word was shown, the participants pressed a button on the mouse indicating whether the word was congruent (left click) or incongruent (right click) with the semantic category presented at the beginning of the trial. Participants were instructed to respond as quickly and correctly as possible.

One possible retrieval strategy individuals could use in theory is to generate informed guesses based on successfully retrieved semantically related items. For example, if an individual cannot recognize the item ‘table’, they may guess that it probably has appeared before because it is related to the category ‘furniture’, which they do clearly remember. Another similar possibility is that an individual might have a sense of familiarity with the item ‘table’, because it is related to the previously encoded semantic category ‘furniture’. This might inflate the amount of hits for congruent items. In order to avoid such possible confounds, each category had three matching words randomly selected for the congruent condition and the remaining three words were randomly assigned to the incongruent condition. Thus, each semantic category had an equal amount of congruent and incongruent word pairs during encoding and test. In this way, differences in recognition performance between congruent and incongruent items could not be due to differences in memory strength for specific categories.

There were 198 congruent-list trials and 198 incongruent-list trials. Together, the study phase lasted 50 min. At the end of this phase, participants were presented with a distraction task in which they solved simple arithmetical problems (additions and subtractions) in order to avoid an active rehearsal of the previously presented words. The distraction task lasted approximately 5 min, which together with the explanations for it and for the subsequent recognition test made for a total time interval of 10 min between encoding and the subsequent test.

Included in the recognition test were a total of 396 Old-word (all items presented at encoding) and 396 New-word trials. The trials were presented in a pseudorandom order for each participant, thus directly avoiding any possible confounds due to order during the test. Words in the Old and New categories were predetermined and the same for all participants. Each of the 792 trials started with a fixation cross in the screen center (1500 ms). All words in the recognition phase were displayed in the middle of the screen, in green and same font and size as the study phase, each for 4000 ms. After each word, participants responded by pressing one of 4 keys according to whether the word was judged to be “sure old,” “guess old,” “guess new,” or “sure new.” The scale graduations were color-coded on the keyboard. Participants were instructed to respond within 4000 ms. Every 50 trials the participants could take a short break. The test phase had a duration of 60 min approximately.

Considering that the retention interval between encoding and test was only 10 min, the paradigm was thus designed to capture only the encoding component and not the consolidation-dependent processes underlying the semantic congruence (schema) effect. Here our distinction between encoding and consolidation is based on the notion of systems consolidation^60^ as a process that includes changes during sleep and over longer time intervals (days, weeks, months, years). Thus a retention delay of ten minutes excludes the possible effects of longer term or sleep related processes such as spindles^61^. However, we do not mean to exclude the possibility of putative initial cellular or other memory consolidation processes taking place within these first 10 minutes^62^.

### Statistical analyses of memory results

ANOVAs (IBM SPSS Statistics 22), with encoding condition (two levels: Congruent vs Incongruent) as a within-participant factor, and age group (two levels: Young and Older participants) as a between-participant factor, were performed on the response rates and reaction times. For all analyses, *α* was set at 0.05. To estimate effect sizes, we used η_p_^2^ and Cohen’s *d* as appropriate. In the case of a participant judging a word sequence during encoding differently than we had predesigned, the sequence was not included (see Table 1 for mean amount of trials included). Participants’ subjective congruence ratings almost always coincided with our experimental design (94%). Given that older participants show lower memory performance for high-confidence responses^63–65^, we specifically ran the tests including only high-confidence responses. Corrected Hit Rates (CHR) were calculated by subtracting the proportion of erroneous ‘old’ responses from the proportion of correct ‘old’ responses. To analyze only high-confidence responses, only high-confidence correct and erroneous responses were included in the CHR. Note that erroneous ‘old’ responses were for words that were not actually presented during encoding, so they were not divided into congruent or incongruent conditions, and so this same false-alarm rate was subtracted from both conditions. For the partial correlation analysis, sample linear correlation coefficients were calculated using the ‘partialcorr’ MATLAB function, using a Student’s *t* distribution for the transformation of the correlation for the *p*-values. Post-hoc t-tests were used when applicable to further investigate any significant interactions detected in the ANOVAs.

**Table 1:** Trials for EEG analyses.

Age	Trial Type	Initial Trials	TF Trials	ERP trials
Young	Congruent	180.00	176.52	168.47
	Incongruent	186.39	183.17	174.83
	Remembered	180.91	178.13	170.70
	Forgotten	118.91	116.78	111.04
	Error	29.00		
Older adults	Congruent	185.04	181.80	160.20
	Incongruent	185.28	181.24	159.04
	Remembered	191.20	186.48	168.44
	Forgotten	133.48	131.68	111.28
	Error	27.48		

Note: For both age groups the mean trials included in each category are shown. The initial trials column shows all trials before removing any trials because of EEG artifacts. Words which were incorrectly classified during encoding (e.g., congruent words classified as incongruent) are shown in the ‘Error’ row and were excluded from the EEG analyses. Guessed ‘old’ responses were not included in the ‘Remembered’ category. Mean trials included in the TF analysis are shown in the ‘TF Trials’ column, and trials included in the ERP analysis are shown under ‘ERP Trials’.

### EEG analysis – Experiment 2

During encoding, electroencephalographic (EEG) activity was acquired with an Easy Cap system by BrainProducts with 32 standard active electrodes. For detecting vertical and horizontal eye movement (VEOG/HEOG), 4 electrodes were used. Impedances were maintained less than 20 kΩ. FCz served as reference and AFz as ground electrode. The sampling rate was at 500 Hz with online high-pass (0.1 Hz) and low-pass (240 Hz) filters. EEGLAB (version 13^66^; and customized MATLAB version 2016b (The MathWorks) tools were used for preprocessing the EEG data offline.

### ERP analysis

For the ERP analysis, a filter appropriate for slow components was used^67,68^. Specifically, the data were filtered offline with a low-pass filter (Hamming window, cut-off frequency at 40 Hz, and the filter order at 166) with the ‘pop_firws’ EEGLAB function, with no additional high-pass filtering. Subsequently, all trials of the encoding phase were epoched and down sampled to 250 Hz. ERPs during the encoding were studied by extracting event-locked EEG epochs of 1600 ms, ending 1500 ms after stimulus onset, with the 100 ms prior to stimulus onset used for the baseline. Subsequently, major atypical artifacts, trials with amplifier saturation, and bad channels were visually identified and removed, (maximum 4 channels, mean = 0.58). Afterwards, blinks and eye movement artifacts were removed with independent component analysis (ICA^66,69^). Finally, bad channels were interpolated. Oz was selected to re-reference the data, as re-referencing to average can mask the effects of EEG differences with a broad distribution across the scalp^70^, such as we expected following previous experiments^5^. EEG trials with a shift exceeding 100 μV were rejected offline.

For the congruence and N400 analysis, only the trials correctly classified during encoding were divided into two separate conditions, ‘Congruent’, and ‘Incongruent’ (including both subsequently remembered and forgotten trials, see Table 1 for mean amount of trials for each condition, including mistakes). For the DM analyses, only trials correctly classified during encoding were divided into two separate conditions, ‘Remembered’, and ‘Forgotten’ (including both congruent and incongruent trials, see Table 1 for mean amount of trials for each condition). For the congruence by memory interaction analyses, only trials correctly classified during encoding were divided into four separate conditions, ‘Congruent Remembered’, ‘Incongruent Remembered’, ‘Congruent Forgotten’, and ‘Incongruent Forgotten’ (see Table 2 for mean amount of trials for each condition). To increase the reliability of the DM analyses and the memory by congruence analyses, only high-confidence correct ‘old’ responses were included in the ´Remembered´ conditions. High confidence and guessed misses (‘new’ responses for words actually presented during the encoding phase) were included in the ´Forgotten´ condition. Three young participants and one older participant were excluded from the analysis due to excessively noisy data or being a low performing outlier (with exceedingly low HR compared to their age group as identified with SPSS using a step of 1.5 × Interquartile Range). Fieldtrip^71^ and customized MATLAB scripts were used for statistical data analysis.

**Table 2:** Trials for EEG analyses included in the congruence by memory interaction analyses.

	Congruent Remembered	Incongruent Remembered	Congruent Forgotten	Incongruent Forgotten
Young	106	65.09	36.43	74.61
Older participants	102.8	65.64	39.80	71.48

Note: For both age groups the mean trials for the congruent remembered, incongruent remembered, congruent forgotten and incongruent forgotten subcategories are shown. Only clean trials included in the ERP analysis as remained after artifact rejection are shown.

To detect reliable differences between the conditions during encoding without imposing any a prioris, the conditions were contrasted using Fieldtrip via a two-tailed non-parametric cluster-based permutation test^72^. Note however that this method does not ensure that the precise boundaries of the cluster detected are exact. All time points between 50 ms and 1500 ms at 27 scalp electrodes (Fp1, Fp2, F7, F3, Fz, F4, F8, FC5, FC1, FC2, FC6, T7, C3, Cz, C4, T8, CP5, CP1, CP2, CP6, P7, P3, Pz, P4, P8, O1, O2) were included in the test. Time points before 50 ms were too early to be considered relevant for the test. For all contrasts, a *t* test was performed for each sample (channel, time). For each permutation, all t scores corresponding to uncorrected *p* values of 0.05 were formed into clusters. The sum of the t scores in each cluster is the ‘mass’ of that cluster and the most extreme cluster mass in each of the sets of tests was recorded and used to estimate the distribution of the null hypothesis. The Monte Carlo estimate was calculated by running random permutations of the condition labels (*n* = 1000) and comparing the cluster statistics found in the real data with that found in the random data. The p-value is thus obtained with the proportion of cluster statistics in the random data exceeding that in the real data. Clusters were formed from significant samples (*p* < 0.05), considering only effects with minimum three significant neighboring channels based on triangulation.

In addition, we ran analyses comparable with previous ERP studies of the N400 and the LPC components, to more thoroughly and comprehensively characterize the ERP differences found. A separate 2×2×2 ANOVA on the mean amplitudes of the N400 component during encoding was conducted, with congruence, memory and age group as factors. The mean amplitudes were taken from 200 to 600 ms, averaging across 4 central parietal electrodes (Cz, CP1, CP2, Pz) where the N400 effect is typically observed (for a review see^27^). A separate 2×2×2 ANOVA on the mean amplitudes of the LPC component during encoding was conducted, with congruence, memory and age group as factors. The mean amplitudes were taken from 600 to 1000 ms, averaging across 3 parietal electrodes (P3, Pz, P4) where the LPC effect is typically observed (see^30^).

### Time-Frequency analysis

For the Time-Frequency (TF) analysis, the data were high-pass (0.5 Hz) and low-pass (Hamming window, bandpass edge at 35 Hz) filtered. Second, all trials of the encoding phase were epoched and down sampled. We extracted event-locked EEG epochs of 4500 ms starting at 2000 ms before the presentation of the first word of the word list. Subsequently, major atypical artifacts, trials with amplifier saturation, and bad channels were visually identified and removed (maximum 1 channel per participant). Otherwise, the preprocessing was conducted as described above for the ERPs.

TF decompositions were conducted from 2 Hz to 30 Hz, from 1000 ms before stimulus onset to 1500 ms after stimulus onset, using convolution on the single-trial time series with complex Morlet wavelets (4 cycles), with steps of 8 ms in the time and 0.22 Hz in the frequency domain. For each condition, power was averaged across trials. A 300 ms baseline correction was applied (from 500 ms before stimulus onset to 200 ms before stimulus onset). The power values thus obtained indicated the relative change as compared to the power during the baseline period, that is, a value of 1 would indicate no change respect to baseline. Note that in the statistical tests and the figures, we subtracted different conditions from each other, so that a value of 0 in those contrasts would indicate no differences between conditions.

To detect reliable differences between the conditions during encoding, the conditions were contrasted using Fieldtrip via a two-tailed non-parametric cluster-based permutation test (Maris and Oostenveld, 2007), on the frequency range from 2 Hz to 30 Hz. To further investigate the observed TF effects for the theta (4–7.5 Hz), alpha (8–13.5 Hz), low beta (14–20.5 Hz) and high beta (21–30 Hz) band, excluding possible edge and/or spillover effects around frequency borders, four separate analyses were run. For all contrasts, a *t* test was performed for each sample (channel, frequency, time), the rest of the analysis was performed as described above for the ERPs.

## Results

### Behavioral findings

#### Experiment 1

##### Main effects of congruence, and age, and a congruence by age interaction, for high-confidence corrected hit rate were found

**Figure 2:**
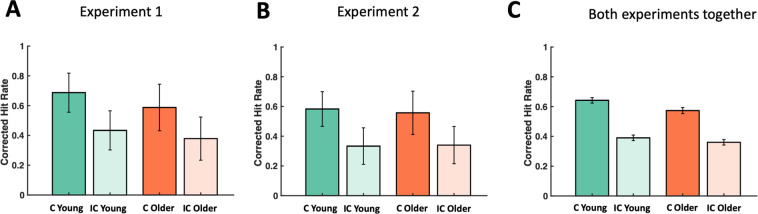
(**A**) CHR for Experiment 1. All conditions were above chance level. There was a main effect of congruence, a main effect of age, and a congruence by age interaction. The increase in CHR due to congruence (CHR for congruent words minus CHR for incongruent words) was greater in the younger participants than in the older participants. (**B**) CHR for Experiment 2. All conditions were above chance level. There was a main effect of congruence, but the effect of age and the congruence by age interaction were not significant. (**C**) CHR for both experiments together. All conditions were above chance level. There was a main effect of congruence, and a congruence by age interaction. The effect of age was not significant. CHR = HR – FA rate (only including high-confidence responses). Error bars indicate SEM.

The proportions of high-confidence ‘Sure’ responses during the recognition phase were analyzed (see Fig. 2A, and Table 3 for hit and false-alarm rates including guesses and high confidence responses). A 2 × 2 ANOVA with the factors congruence and age revealed a significant main effect of congruence (F_(1,55)_ = 496.26, p < 0.001, η_p_^2^ = 0.90), driven by higher CHR for congruent words, than for incongruent words. There was also a significant main effect of age (F_(1,55)_ = 4.90, p = 0.031, η_p_^2^ = 0.08), with higher CHR for younger, than for older participants. Importantly, a significant congruence by age interaction effect was also revealed (F_(1,55)_ = 4.94, p = 0.030, η_p_^2^ = 0.08). The increase in CHR due to congruence (CHR for congruent words minus CHR for incongruent words) was greater in the younger participants than in the older participants.

**Table 3:** Memory performance.

Group	Category	All Responses	Sure Responses
**Experiment 1**			
Young	Congruent HR	0.84 (0.02)	0.72 (0.02)
	Incongruent HR	0.64 (0.02)	0.47 (0.03)
	False Alarm rate	0.09 (0.02)	0.04 (0.01)
Older adults	Congruent HR	0.75 (0.03)	0.64 (0.04)
	Incongruent HR	0.56 (0.03)	0.43 (0.04)
	False Alarm rate	0.09 (0.02)	0.05 (0.01)
**Experiment 2**			
Young	Congruent HR	0.77 (0.02)	0.62 (0.03)
	Incongruent HR	0.57 (0.02)	0.37 (0.03)
	False Alarm rate	0.09 (0.02)	0.04 (0.01)
Older adults	Congruent HR	0.74 (0.03)	0.63 (0.03)
	Incongruent HR	0.54 (0.03)	0.41 (0.04)
	False Alarm rate	0.11 (0.02)	0.07 (0.02)

Note: Mean and SEM for Hit Rates (HR) and False-Alarm rates for both experiments and age groups are shown. Under the ‘All Responses’ column both high confidence and low confidence ‘Old’ responses (sure and guess responses) are shown. Under the ‘Sure Responses’ column only high confidence responses are included.

##### Main effect of congruence for RT

For participants’ reaction times during the encoding phase, we found a main effect of congruence (*F*_(1,55)_ = 42.40, *p* < 0.001, η_p_^2^ = 0.44), participants were slower at identifying incongruent words (see Table 4). However, age did not reach significance level (*F*_(1,55)_ = 2.29, *p* = 0.136, η_p_^2^ = 0.04), and there was no significant congruence by age interaction (*F*_(1,55)_ = 0.59, *p* = 0.447, η_p_^2^ = 0.01).

**Table 4:** Reaction times for the semantic matching encoding task.

		Congruent	Incongruent
Behavioral Group	Young	845.14 (42.12)	899.80 (48.74)
	Older adults	933.38 (43.14)	1002.63 (45.56)
EEG Group	Young	855.04 (50.62)	888.15 (52.34)
	Older adults	964.75 (35.82)	1015.76 (39.95)

Note: Mean (SEM) reaction times in ms. Here we included only trials in which the participants correctly classified the word pairs. The anova showed a main effect of congruence (p < 0.001), participants were slower to classify incongruent words than congruent words.

#### Experiment 2

##### Main effect of congruence for high-confidence CHR

In Experiment 2, the proportions of high-confidence ‘Sure’ responses during the recognition phase were analyzed, again, in a 2 × 2 ANOVA (see Fig. 2B, and Table 3 for hit and false-alarm rates including guesses and high confidence responses). This analysis only showed a significant main effect for congruence (*F*_(1,46)_ = 292.01, *p* < 0.001, η_p_^2^ = 0.86) with higher CHR for congruent words (mean 0.57, SEM 0.02), than for incongruent words (mean 0.34, SEM 0.03). In contrast to the behavioral experiment (Experiment 1), there was no main effect of age (*F*_(1,46)_ = 0.075, *p* = 0.785, η_p_^2^ < 0.01), and no congruence by age interaction effect (*F*_(1,46)_ = 1.39, *p* = 0.244, η_p_^2^ = 0.03). The increase in CHR due to congruence (CHR for congruent words minus CHR for incongruent words) was not significantly greater in the younger participants than in the older participants (*t*_(52)_ = 1.55, *p* = 0.13, Cohen’s *d* = 0.42).

##### Main effect of congruence for RT

For participants’ reaction times during the encoding phase, there was a main effect of congruence (*F*_(1,46)_ = 22.42, *p* < 0.001, η_p_^2^ = 0.33), participants were slower at identifying incongruent words (see Table 4). However, age did not reach significance level (*F*_(1,46)_ = 3.59, *p* = 0.06, η_p_^2^ = 0.07), and there was no significant congruence by age interaction (*F*_(1,46)_ = 1.02, *p* = 0.319, η_p_^2^ = 0.02). This result on reaction times replicated the results of the behavioral experiment and is in line with the recognition memory performance (i.e. no age effect and no interaction).

#### Analysis of both experiments

There were no differences between age groups and experimental groups in performance in the short arithmetic task, which was presented between encoding and retrieval (all p > 0.25). Mean performance for the arithmetic task in each of the four behavioral and age groups was above 94%. There were no differences between any of the groups in their performance in the encoding task either between age groups or experimental groups (all p > 0.16). Mean performance for the encoding task in each of the four behavioral and age groups was equal to or above 94%.

##### Main effect of congruence, experiment group, and a congruence by age interaction, for high-confidence CHR

A separate 2 × 2 × 2 ANOVA on high-confidence ‘Sure’ responses during the recognition phase, with congruence, age group, and experimental group as factors, including all the participants from both experiments, revealed a significant main effect of congruence (*F*_(1,101)_ = 757.54, *p* < 0.001, η_p_^2^ = 0.88); a marginal effect of age (*F*_(1,101)_ = 3.14, *p* = 0.079, η_p_^2^ = 0.030); and a main effect of experiment (*F*_(1,101)_ = 7.00, *p* = 0.010, η_p_^2^ = 0.065), with lower CHR in the EEG group (see Fig. 2C). Importantly, there was a significant congruence by age interaction effect (*F*_(1,101)_ = 5.38, *p* = 0.022, η_p_^2^ = 0.05), driven by a stronger congruence effect in the younger participants as compared to older participants. Finally, there were no other interactions between experimental group and age or congruence (all *p* > 0.165).

##### Main effect of congruence, and age, for RT

There was a main effect of congruence for participants’ reaction times (*F*_(1,101)_ = 63.37, *p* < 0.001, η_p_^2^ = 0.38), participants were slower at identifying incongruent words (see Table 4). There was also a significant main effect of age (*F*_(1,101)_ = 5.72, *p* = 0.019, η_p_^2^ = 0.05), younger participants were faster, but there was no significant congruence by age interaction (*F*_(1,101)_ = 1.55, *p* = 0.216, η_p_^2^ = 0.02). There was no significant main effect of EEG group (*F*_(1,101)_ = 0.67, *p* = 0.796, η_p_^2^ = 0.1), and no other interactions between EEG group and age or congruence (all *p* > 0.129).

### EEG findings

#### ERP Cluster analysis

##### Main effect of congruence, and a congruence by age interaction

**Figure 3:**
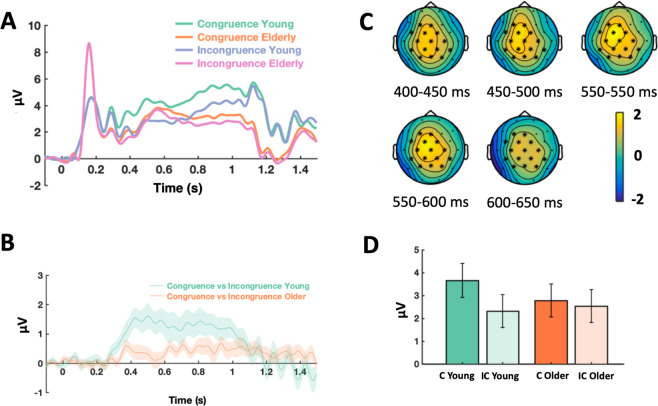
ERP congruence effect cluster. (**A**) Mean amplitude of the congruence by age ERP interaction averaged across the significant electrodes found in the cluster. The time window of the significant differences detected in the cluster was from 500 to 600 ms aprox. (**B**) The difference wave of the congruent minus the incongruent condition in the young group was contrasted against the same difference wave in the older group, in the cluster analysis. For the figure, the mean was calculated from the grand averages across the 15 electrodes (F3, Fz, FC5, FC1, FC2, C3, Cz, C4, CP5, CP1, CP2, CP6, P3, Pz, P4) of the cluster. (**C**) ERP Topoplots (mean amplitude in *µV*) of the significant cluster of the congruence by age interaction. Electrodes where significant differences were detected corresponding to the interaction are highlighted with asterisks. The difference wave of the congruent minus the incongruent condition in the young group was contrasted with the same difference wave in the older group in the cluster analysis. (**D**) Barplot showing the mean amplitude across the electrodes of the significant cluster of the congruence by age interaction, from the significant time-range (484 to 600 ms), with the congruent (c) and incongruent condition (ic) shown for the groups of young and older adults.

A Monte Carlo cluster-based permutation test was performed on the data of young and older participants grouped together, from 50 ms to 1500 ms after word onset, which detected a positive cluster due to congruence during encoding, from approximately 300 ms to 1200 ms, with a broad central topography including frontal, central and parietal electrodes (*p* = 0.002, congruent vs incongruent, see Table 5). Next, the effect of congruence between young and older participants was contrasted in the same way (congruent minus incongruent in young participants vs congruent minus incongruent in older participants), and a positive cluster was found, from approximately 500 ms to 600 ms, with a broad central topography including frontal, central and parietal electrodes (*p* = 0.012; see Fig. 3). As such, we found a main effect of congruence, as well as an interaction between age and congruence which was not observed at the behavioral level in the same subjects.

**Table 5:** Significant Clusters.

	Cluster	Duration (ms)	Frequency range (Hz)	Electrodes	p
**ERP** (positive going)	**Congruence** contrast	316–1184	—	F3 Fz F4 FC5 FC1 FC2 FC6 C3 Cz C4 T8 CP5 CP1 CP2 CP6 Pz P4	0.002
	**Congruence by age** interaction	484–600	—	F3 Fz FC5 FC1 FC2 C3 Cz C4 CP5 CP1 CP2 CP6 P3 Pz P4	0.012
	**DM** contrast	220–1496	—	F3 Fz F4 FC5 FC1 FC2 FC6 T7 C3 Cz C4 T8 CP5 CP1 CP2 CP6 Pz P4	0.002
	**DM by age** interaction	380–1256	—	F3 Fz F4 FC5 FC1 FC2 FC6 T7 C3 Cz C4 T8 CP5 CP1 CP2 CP6 Pz P4	0.002
**TF** (relative power decrease)	**Congruence** contrast	50–1440	2–25.56	F3 Fz F4 FC5 FC1 FC2 FC6 T7 C3 Cz C4 T8 CP5 CP1 CP2 CP6 P3 Pz P4	0.002
	**Congruence by age** interaction	728–1072	4.89–13.56	F3 Fz F4 FC5 FC1 FC2 FC6 T7 C3 Cz C4 T8 CP5 CP1 CP2 CP6 P3 Pz P4	0.030
	**DM**	608–1344	5.77–28.00	F3 Fz F4 FC5 FC1 FC2 FC6 T7 C3 Cz C4 T8 CP5 CP1 CP2 CP6 P3 Pz P4	0.004
	**Congruence by Memory** interaction	480–736	11.33–27.11	F3 Fz F4 FC5 FC1 FC2 FC6 T7 C3 Cz C4 T8 CP5 CP1 CP2 CP6 P3 Pz P4	0.026

Note: Contrasts for each cluster are indicated as detected by the two-tailed non-parametric cluster-based permutation tests. ERP and TF clusters are presented. Congruence contrasts (congruent minus incongruent), congruence by age interaction contrasts (Congruence contrast in young group minus congruence contrast in older participant group), DM contrasts (remembered minus forgotten) and DM by age interaction contrasts (DM contrast in young group minus DM contrast in older participant group) are shown. The duration in ms and the list of electrodes which were detected in the cluster are shown for each contrast, together with the significance level, although the exact limits of each cluster as returned by the test are only aproximates.

##### Main DM effect, and a DM by age interaction

**Figure 4:**
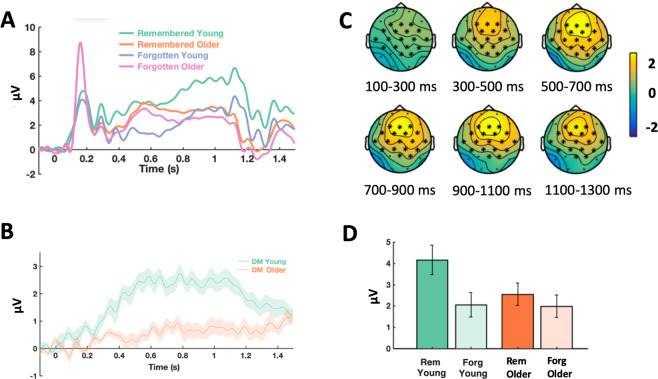
Memory by Age ERP interaction cluster. (**A**) Mean amplitude of the memory by age ERP interaction averaged across the significant electrodes found in the cluster. The time windows of the significant differences detected in the cluster was from 400 to 1300 ms aprox. (**B**) The difference wave of the remembered minus the forgotten condition in the young group was contrasted against the same difference wave in the older group, in the cluster analysis. For the figure, the mean was calculated from the grand averages across the 18 electrodes (F3, Fz, F4, FC5, FC1, FC2, FC6, T7, C3, Cz, C4, CP5, CP1, CP2, CP6, Pz, P4) of the cluster. (**C**) ERP Topoplots (mean amplitude in *µV*) of the significant cluster of the memory by age interaction. Electrodes where significant differences were detected corresponding to the interaction are highlighted with asterisks. The difference wave of the congruent minus the incongruent condition in the young group was contrasted with the same difference wave in the older group in the cluster analysis. (**D**) Barplot showing the mean amplitude across the electrodes of the significant cluster of the memory by age interaction, from the significant time-range (380–1256 ms), with the remembered (rem) and forgotten condition (forg) shown for the groups of young and older adults.

The cluster-based permutation test, on the data of young and older participants grouped together, from 50 ms to 1500 ms after word onset, revealed a positive cluster of Differences due to Memory, from approximately 200 ms to 1500 ms, with a broad central topography including frontal, central and parietal electrodes (*p* = 0.002; DM effect; remembered vs forgotten, see Table 5). When the DM effect between young and older participants was contrasted in the same way (remembered minus forgotten in young participants vs remembered minus forgotten in older participants), significant differences in three different positive clusters were found similar to the congruence effects, from approximately 400 ms to 1300 ms, all with a broad central topography including frontal, central and parietal electrodes (*p* = 0.002; see Fig. 4).

##### Behavioral EEG correlations

**Figure 5:**
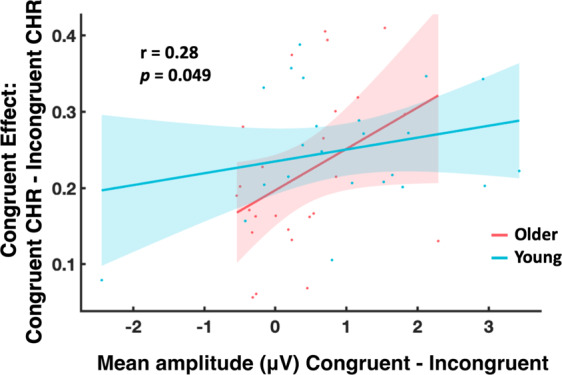
Relationship between congruence effect and ERPs. Inter-individual correlation between the ERP difference wave (mean amplitude in μV) of the congruent condition minus the incongruent condition, averaged across time (316–1184 ms after stimulus onset) and the central and parietal electrodes of the cluster (C3, Cz, C4, CP5, CP1, CP2, CP6, P3, Pz, P4), with the difference resulting from congruent high-confidence CHR minus incongruent high-confidence CHR, across participants from both age groups controlling for age. The lines show the fit of a generalized linear model to the data for each age group separately, with the 95% confidence bounds displayed. The ERP congruent minus incongruent difference wave predicted the difference in congruent memory performance across individuals in both groups controlling for age (Partial correlation; r = 0.28, p = 0.049).

To investigate whether the congruence effect directly relates to neural activity, and test for possible individual correlations between the mean amplitude in the congruence effect cluster and the memory results, within groups, a partial correlation (controlling for age) was run (see Fig. 8). The first variable in the correlation was the behavioral advantage for congruent memories (congruent high-confidence CHR minus incongruent high-confidence CHR). The second variable was the neural activity associated to the congruent condition (Congruent ERP minus Incongruent ERP) measured at the central and parietal electrodes (C3, Cz, C4, CP5, CP1, CP2, CP6, P3, Pz, P4), where congruence differences are typically observed^27^, and that were indeed found in the ERP cluster associated to congruence (Congruent ERP minus Incongruent ERP), from 316 ms to 1184 ms. Across all participants, and controlling for age, a significant correlation was revealed (r = 0.28, *p* = 0.049): the greater the difference between the ERP in the congruent condition vs the incongruent condition across the central and parietal electrodes, the greater the difference between the congruent high-confidence CHR and incongruent high-confidence CHR. Post hoc analysis revealed no statistically significant difference for the independent samples of young vs. older participants (p > 0.3; see Fig. 5).

#### N400 ERP analysis

We performed additional analyses to more thoroughly characterize the ERP effects by studying the role of the N400 in the observed cluster.

##### N400 mean amplitude ANOVA: main effect of congruence, main effect of memory, congruence by age interaction, memory by age interaction, and a congruence by memory interaction

A separate 2 × 2 × 2 ANOVA on the mean amplitudes of the N400 component during encoding was conducted, with congruence, memory and age group as factors. The mean amplitudes were taken from 200 to 600 ms, averaging across 4 central parietal electrodes (Cz, CP1, CP2, Pz) where the N400 effect is typically observed^27^. The ANOVA revealed a significant main effect of congruence (*F*_(1,46)_ = 15.34, *p* < 0.001, η_p_^2^ = 0.25); a significant main effect of memory (*F*_(1,46)_ = 45.94, *p* < 0.001, η_p_^2^ = 0.50); and a non-significant effect of age (*F*_(1,46)_ = 3.23, *p* = 0.079, η_p_^2^ = 0.07), with ERPs being more positive overall for congruent and remembered words. There was a significant congruence x age interaction (*F*_(1,46)_ = 2.26, *p* = 0.026, η_p_^2^ = 0.103); with the decrease due to age in ongoing positivity being greater for congruent words (mean difference 2.28; 95% CI [0.27, 4.29]) than for incongruent words (mean difference 1.41; 95% CI [−0.55, 3.39]); i.e., age effects were greater for congruent words than for incongruent words. There was also a significant memory × age interaction (*F*_(1,46)_ = 10.20, *p* = 0.003, η_p_^2^ = 0.181), with the decrease due to age in ongoing positivity being greater for remembered words (mean difference 2.20; 95% CI [0.17, 4.24]) than for forgotten words (mean difference 1.19; 95% CI [−0.71, 3.09]); i.e., age effects were greater for remembered words than for forgotten words. This analysis confirmed the results of the cluster-based permutation tests for the N400 component. In addition, there was a significant congruence × memory interaction (*F*_(1,46)_ = 4.43, *p* = 0.041, η_p_^2^ = 0.09), with the N400 congruence effect being greater for remembered words (mean difference 0.92; 95% CI [0.56, 1.28]) than for forgotten words (mean difference 0.34; 95% CI [−0.17, 0.85]). The main effect of age was non-significant (*F*_(1,46)_ = 3.23, *p* = 0.079, η_p_^2^ = 0.066), as was the congruence x memory x age interaction (*F*_(1,46)_ = 0.02, *p* = 0.896, η_p_^2^ < 0.001).

##### Behavioral correlations with N400 mean amplitude

To investigate whether the congruence effect directly relates to the N400 component, and test for possible individual correlations between the N400 amplitude and the memory results, within groups, a partial correlation (controlling for age) was run. The first variable in the correlation was the behavioral advantage for congruent memories (congruent high-confidence CHR minus incongruent high-confidence CHR). The second variable was the N400 component measured as described above (Incongruent ERP minus Congruent ERP) measured at central and parietal electrodes (Cz, CP1, CP2, Pz), from 200 to 600 ms^27^. Across both age groups, and controlling for age, a significant correlation was revealed (r = −0.32, *p* = 0.031): the greater the N400, the greater the difference between congruent high-confidence CHR and incongruent high-confidence CHR. Post hoc analysis revealed no statistically significant differences for the independent samples of young vs. older participants (p > 0.3).

#### LPC ERP analysis: main effect of memory, main effect of age, memory by age interaction, and a congruence by memory interaction

We performed additional analyses to more thoroughly characterize the ERP effects by studying the role of the LPC in the observed cluster. A separate 2 × 2 × 2 ANOVA on the mean amplitudes of the LPC component during encoding was conducted, with congruence, memory and age group as factors. The mean amplitudes were taken from 600 to 1000 ms, averaging across 3 parietal electrodes (P3, Pz, P4) where the LPC effect is typically observed. The ANOVA revealed a non-significant main effect of congruence (*F*_(1,46)_ = 3.34, *p* = 0.074, η_p_^2^ = 0.07); a significant main effect of memory (*F*_(1,46)_ = 40.93, *p* < 0.001, η_p_^2^ = 0.47); and a significant effect of age (*F*_(1,46)_ = 6.09, *p* = 0.017, η_p_^2^ = 0.12), with ERPs being more positive overall for remembered words and younger participants. There was a non-significant congruence x age interaction (*F*_(1,46)_ = 0.01, *p* = 0.925). There was also a significant memory x age interaction (*F*_(1,46)_ = 10.80, *p* = 0.002, η_p_^2^ = 0.19), with the decrease due to age in ongoing positivity being greater for remembered words (mean difference 2.17; 95% CI [0.74, 3.59]) than for forgotten words (mean difference 1.36; 95% CI [−0.02, 2.74]); i.e., aging effects were greater for remembered words than for forgotten words. In addition, there was a significant memory × congruence interaction (*F*_(1,46)_ = 7.07, *p* = 0.011, η_p_^2^ = 0.13), with the LPC memory effect being greater for congruent words (mean difference 1.02; 95% CI [0.64, 1.40]) than for incongruent words (mean difference 0.49; 95% CI [0.20, 0.78]). The congruence ×memory × age interaction was non-significant (*F*_(1,46)_ = 3.32, *p* = 0.075).

#### Time-Frequency Cluster Analysis

##### Main effect of congruence, and a congruence by age interaction

**Figure 6:**
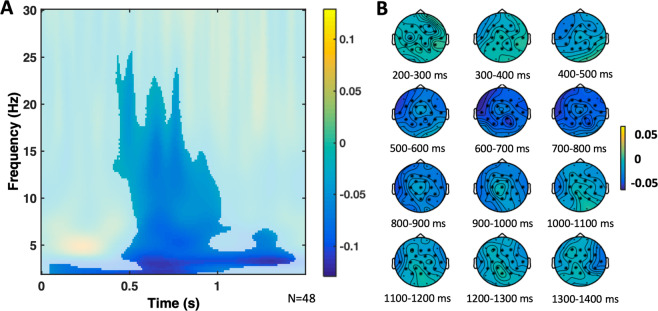
Congruence TF effect cluster. (**A**) Relative power change of the congruence TF effect averaged across the significant electrodes found in the cluster. The significant time window was aprox. from 50 ms to 1440 ms, and the significant frequency window from 2 Hz to 6 Hz. The difference between the congruent minus the incongruent condition was contrasted in the cluster analysis, including both the young and older groups. For the figure, the mean was calculated from the grand averages across the 19 electrodes (F3, Fz, F4, FC5, FC1, FC2, FC6, T7, C3, Cz, C4, T8, CP5, CP1, CP2, CP6, P3, Pz, P4) of the cluster. (**B**) TF power topoplots of the significant cluster of the congruence effect, in the significant TF window. Electrodes in the cluster are highlighted with asterisks.

**Figure 7:**
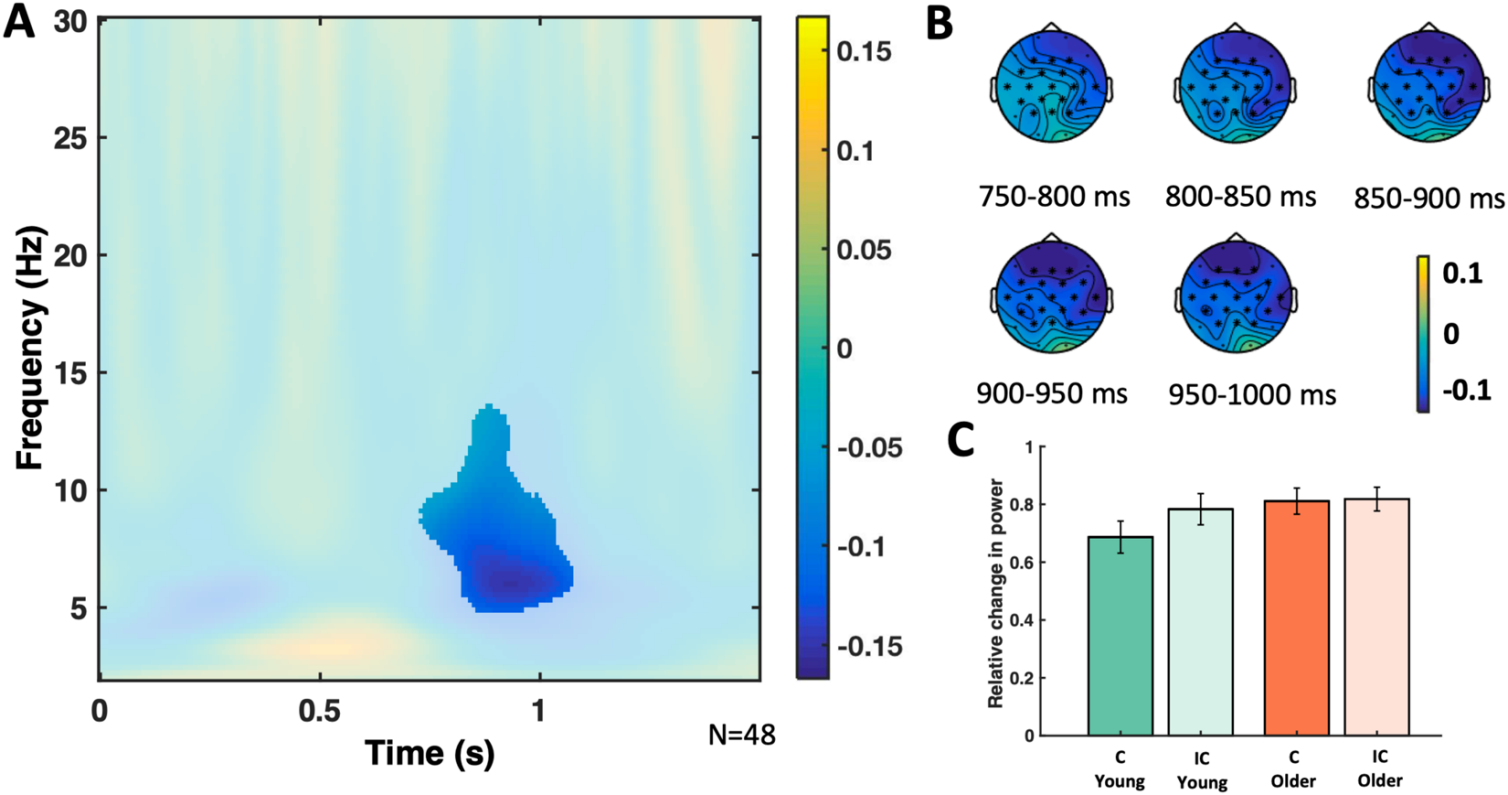
Congruence by Age TF interaction cluster. (**A**) Relative power change of the congruence by age TF interaction across the significant electrodes found in the cluster. The significant time window was approximately from 700 ms to 1100 ms, and the significant frequency window was from 5 Hz to 14 Hz. The difference in relative power of the congruent minus the incongruent condition in the young group was contrasted against the same difference in the older group, in the cluster analysis. For the figure, the mean was calculated from the grand averages across the 19 electrodes (F3, Fz, F4, FC5, FC1, FC2, FC6, T7, C3, Cz, C4, T8, CP5, CP1, CP2, CP6, P3, Pz, P4) of the cluster. (**B**) TF power topoplots of the significant cluster of the congruence by age interaction, in the significant TF window. Electrodes with significant differences corresponding to the interactions are highlighted with asterisks. (**C**) Barplot showing the relative power change across the electrodes of the significant cluster of the congruence by age interaction, in the significant TF window, with the congruent (c) and incongruent condition (ic) shown for the groups of young and older adults.

A Monte Carlo cluster-based permutation test was run on the TF data of young and older participants grouped together (see Table 5), from 50 ms to 1500 ms after word onset, and from 2 to 30 Hz. It revealed significant differences due to congruence (congruent vs incongruent) with a negative cluster, with a broad central topography, approximately from 2 Hz to 26 Hz, from 50 ms to 1440 ms (*p* = 0.002; see Fig. 6). In a next step, the differential effect of congruence between young and older participants was contrasted in the same way (congruent minus incongruent in young participants vs congruent minus incongruent in older participants). This analysis revealed significant differences (see Fig. 7) with a negative cluster, with a broad central topography, approximately from 5 Hz to 14 Hz, from 700 ms to 1100 ms after stimulus onset (*p* = 0.030).

##### Main effect of DM, and a memory by congruence interaction

**Figure 8:**
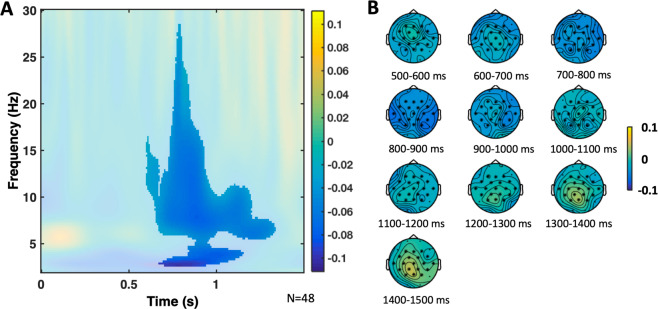
DM TF effect cluster. (**A**) Relative power change of the DM TF effect averaged across the significant electrodes found in the cluster. The significant time window was approximately from 600 ms to 1300 ms, and the significant frequency window was from 6 Hz to 28 Hz. The difference between the remembered minus the forgotten condition was contrasted in the cluster analysis, including both the young and older groups. For the figure, the mean was calculated from the grand averages across the 19 electrodes (F3, Fz, F4, FC5, FC1, FC2, FC6, T7, C3, Cz, C4, T8, CP5, CP1, CP2, CP6, P3, Pz, P4) of the cluster. (**B**) TF power topoplots of the significant cluster of the DM effect, in the significant TF window. Electrodes in the cluster are highlighted with asterisks.

The cluster-based permutation test, on the data of young and older participants grouped together, from 50 ms to 1500 ms after word onset, and from 2 to 30 Hz, revealed significant differences with a negative cluster due to memory (*p* = 0.004; remembered vs forgotten, see Fig. 8, and Table 5), approximately from 600 ms to 1300 ms, approximately 6 Hz to 28 Hz. When we contrasted for differences due to memory between young and older participants in the same way (remembered minus forgotten in young participants vs remembered minus forgotten in older participants), we did not find any significant differences. In addition, a test was run for the congruence × memory interaction, that is, the difference due to memory in the congruent condition was contrasted with the difference due to memory in the incongruent condition. This analysis revealed significant differences with a negative cluster, with a broad central topography, approximately from 11 Hz to 27 Hz, from 500 ms to 700 ms after stimulus onset, with greater negativity for the DM contrast for congruent words than for incongruent words (*p* = 0.026).

There was no significant correlation between the mean power in the congruence effect TF cluster with the behavioral advantage for congruent memories (congruent high-confidence CHR minus incongruent high-confidence CHR), controlling for age.

##### Separate analyses for theta, alpha, and low beta band

To further investigate the observed TF effects for the theta (4–7.5 Hz), alpha (8–13.5 Hz), low beta (14–20.5 Hz) and high beta (21–30 Hz) band, four separate analyses were performed. As expected and largely confirming the initial cluster-based analyses, they revealed main effects of congruence (theta, alpha and low beta) and significant interactions between congruence and age (theta and alpha). Table 6 gives a detailed overview including frequency bands, time points and sensors detected in the clusters. Finally, DM analyses were computed for the separate frequency bands. Again, largely in agreement with the initial cluster analysis, they revealed main effects of DM (theta, alpha, low beta), as well as a congruence by memory interaction for low beta only – see Table 6. There were no significant effects in the high beta frequency band (Table 6).

**Table 6:** Significant TF Clusters for theta, alpha, and low-beta frequency bands.

	Cluster (relative power decrease)	Duration (ms)	Frequency range (Hz)	Electrodes	p
**Theta**	**Congruence** contrast	456–1424	4–7.56	F3 Fz F4 FC5 FC1 FC2 FC6 T7 C3 Cz C4 T8 CP5 CP1 CP2 CP6 P3 Pz P4	0.002
	**Congruence by age** interaction	776–1072	4.89–7.56	F3 Fz F4 FC5 FC1 FC2 FC6 T7 C3 Cz C4 T8 CP5 CP1 CP2 CP6 P3 Pz P4	0.034
	**DM**	672–1344	5.78–7.56	F3 Fz F4 FC5 FC1 FC2 FC6 T7 C3 Cz C4 T8 CP5 CP1 CP2 CP6 P3 P4	0.010
**Alpha**	**Congruence** contrast	408–1016	8–13.56	F3 Fz F4 FC5 FC1 FC2 FC6 T7 C3 Cz C4 T8 CP5 CP1 CP2 CP6 P3 Pz P4	0.002
	**Congruence by age** interaction	728–1032	8–13.56	F3 Fz F4 FC5 FC1 FC2 FC6 T7 C3 Cz C4 T8 CP5 CP1 CP2 CP6 P3 Pz P4	0.028
	**DM**	608–1184	8–13.56	F3 Fz F4 FC5 FC1 FC2 FC6 T7 C3 Cz C4 T8 CP5 CP1 CP2 CP6 P3 Pz P4	0.002
**Low-Beta**	**Congruence** contrast	416–848	14–20.44	F3 Fz F4 FC5 FC1 FC2 FC6 T7 C3 Cz C4 T8 CP5 CP1 CP2 CP6 P3 Pz P4	0.004
	**DM**	728–904	14–20.44	F3 Fz F4 FC5 FC1 FC2 FC6 T7 C3 Cz C4 T8 CP5 CP1 CP2 CP6 P3 Pz P4	0.008
	**Congruence by Memory** interaction	480–736	14–20.44	F3 Fz F4 FC5 FC1 FC2 FC6 T7 C3 Cz C4 T8 CP5 CP1 CP2 CP6 P3 Pz P4	0.002

Note: TF Contrasts for each cluster as obtained after running tests on theta, alpha, and low beta frequency bands separately, are indicated as detected by the two-tailed non-parametric cluster-based permutation tests. Congruence contrasts (congruent minus incongruent), congruence by age interaction contrasts (Congruence contrast in young group minus congruence contrast in older participant group), DM contrasts (remembered minus forgotten), DM by age interaction contrasts (DM contrast in young group minus DM contrast in older participant group), and congruence by DM interaction contrasts (DM contrast for congruent words minus DM contrast for incongruent words) are shown. The duration in ms, the frequency range in Hz, and the list of electrodes which were returned by the computation for each cluster, are shown together with the significance level.

## Discussion

We investigated the neural processes underlying the semantic congruence effect and its relationship with healthy aging. Our results demonstrate that semantic congruence boosts long-term memory in both age groups. At the neural level, there were age-related differences in post-stimulus neural activity, including large differences in ERP amplitude and differences in the relative power of brain oscillations in the theta-alpha and low beta range. Importantly, ERP differences associated with congruent semantic matches across central and parietal electrodes predicted the increases in memory performance for congruent items across participants (i.e., people with greater congruence ERPs showed enhanced semantic congruence effects, see Fig. 5). Thus, our results suggest that semantic congruence increases long-term memory across the life-span, and, at the neural level, this behavioral effect could be linked to ERPs and neural oscillations that were previously associated with semantic processing.

In accordance with a wealth of previous studies^1–6^, and therefore as expected, congruent items were better remembered than incongruent items. At first glance, this congruence effect declined with age, but the interaction between age and congruence was only significant across both experiments, and in the behavioral group alone (Fig. 2). More specifically, in Experiment 1 (behavior), we found a main effect of age on memory, as well as an interaction with congruence, whereas in Experiment 2 (EEG) we did not find a main effect of age or an interaction of age and congruence. Despite our prediction of a clear age-related decline in the congruence effect, our observations are in line with a previous EEG study, which included 30 young and 28 older participants^50^. It suggests a relatively small effect size that requires a large enough sample in order to detect an interaction with age (further supported by our analysis across Experiment 1 and 2). However, the size of the congruence by age interaction effect in Experiment 1 as measured by η_p_^2^ can be considered, according to Cohen^73^, to be of medium size, and in Experiment 2 the effect size of this interaction would technically be considered not medium but small, although not statistically significant.

Alternatively, the absence of a clear interaction between age and congruence at the behavioral level in Experiment 2 could relate to interindividual differences^74^. Indeed, older people vary in their degree of cognitive abilities, ranging from those without any detectable cognitive impairments to individuals close to MCI^75^. We did not screen our participants with a comprehensive neuropsychological battery that would allow a finer discrimination of cognitive aging between the older participant groups (i.e. Experiment 1 vs Experiment 2). However, no significant differences in the MoCA scores or in the performance on the arithmetic distraction task, or any overall difference in the behavioral task itself, were found between the two groups of older adults. If the older participants in Experiment 2 were more fit at a cognitive level, then we would have expected differences in their MoCA scores; however, it is possible that MoCA is not sensitive enough here.

To investigate the overall behavioral effects in both experiments, an ANOVA with experimental group as a factor was conducted. The purpose here was to test for effects in the largest possible sample. As noted above, this analysis revealed an interaction between age and congruence but no significant effect of experimental group or interaction with experimental group was detected. However, these results must be interpreted with caution since null effect findings do not provide evidence that such a difference (between experimental groups) did not actually exist, especially concerning the absence of a main effect of age or interactions with age in Experiment 2. Together, our findings provide only weak evidence that the congruence effect is impaired in the group of older participants. Therefore, the congruence effect might be well preserved in healthy aging. This point, however, needs to be further addressed in future studies ideally including larger sample sizes and different stimulus material.

It is important to note that the advantage for congruent items in our results cannot be explained by a better memory of the category instead of the word item, since each of the categories was equally paired with both congruent and incongruent words. Previous studies have shown how semantic congruence increases both memories for semantically related items^5^ and episodic details^1^, suggesting that semantic congruence does enhance both the specific episodic and the semantic information content of memories. Future studies might help to further clarify this issue.

Reaction times were faster for the accurate classifications of congruent words as compared to incongruent words (see Table 4). This effect appeared consistently across both age groups and is in line with the semantic priming literature^76^. It can be explained with models assuming that top-down connections allow high-level contextual expectancies to affect perception by preparing the visual system before the stimulus even arrives, i.e., a pre-selection of possible congruent words in semantic memory^77^. This suggests that incongruent stimuli are more difficult to process than congruent stimuli, which argues against the otherwise possible explanation that the congruent memory increase was due to additional effort or difficulty during encoding.

At the neural level, the overall ERP differences between congruent vs incongruent items (Fig. 3A) confirms our prediction. It may reflect the timing of memory processes and their modulation by the configuration of activity in memory. In other words, they might represent how semantic congruence facilitates rapid encoding. The age-related reduction in the ERPs (Fig. 3B) are suggestive of an impairment of such rapid online encoding processes. Indeed, the congruence related ERP differences predicted the behavioral advantage for congruent memories (Fig. 5). In agreement with a previous study that found an interaction between congruence and memory in the ERPs^5^, this further supports the notion that the congruence-related ERPs reflect processes involved in the formation of long-term memories. One relatively simple explanation would be that the congruent categories pre-activate nodes related to the subsequent item, which then facilitates the activation of concept nodes related to the subsequent congruent item^78^. This, in turn, would facilitate the encoding process, and the probability of the word being subsequently recognized. How such processes might influence episodic encoding, is not entirely clear^11^. It is important to note, however, that the memory differences between congruent and incongruent words found here cannot be explained by a general conceptual familiarity with the semantic category, or an unspecific conceptual priming or facilitation process during retrieval. The paradigm was designed to control for such confounds, by including three congruent words and three incongruent words for each semantic category.

The correlation analysis shows that individual differences in the congruence effect (i.e. improved memory) vary as a function of ERP congruence differences (see Fig. 5). Interestingly, this correlation was significant across young and older participants suggesting one underlying mechanism of congruence encoding that may continuously change with age and/or other associated factors such as learning strategies or structural brain integrity (which have not been investigated here). However, this hypothesis needs to be addressed in a longitudinal study or a design that includes a more equally distributed age range across the life-span.

Brain oscillations provide insights into semantic processes carried out during encoding^43^. Specifically, alpha and beta power decreases during congruent (vs incongruent) items (Fig. 6), as in our study, may reflect a more successful encoding possibly due to their relation to semantic deep processing, which increases the DM^10^. At the neural level, such desynchronization may be associated with an increase of information processing capability within local cell assemblies^42^. Beta oscillations have also been related to the maintenance of information about recent events that facilitates integrating inputs into a larger representation in memory^41^. More specifically, alpha-band oscillations have been posited to reflect the key process of selective access to long-term knowledge stores, which allows the semantic orientation^44^ that is necessary to form semantic matches. Our significant interaction between congruence and age (Fig. 7) suggests that older adults may have difficulties with such cognitive processes, which might, at a certain stage, impair their memory performance.

Theta oscillations play a key role in the encoding and retrieval of episodic memories^47–49,79–81^. In our study, theta power decreases were associated with processing congruent items, further suggesting a functional role of theta in the semantic congruence effect. While this is, generally speaking, in line with previous work, the direction of the effect is opposite to what has been reported recently^50^. In fact, Crespo-Garcia *et al*.^50^ could show increased theta power for semantically related face-location associations, which, similar to our findings, changed depending on age. While there are several differences between both study designs (including stimulus material and task), this opposite pattern in theta activity is compatible with the view that neural oscillations during encoding depend on perceptual and cognitive processes of the encoding task and their relation to the subsequent memory test^43^.

Changes in theta-alpha and low beta oscillations, which we observed over frontal brain regions (Fig. 6), might also reflect inhibitory processes. Specifically, the “schema-linked interactions between medial prefrontal and temporal regions” (SLIMM) framework^19^ suggests that the medial prefrontal cortex detects congruence with already existing neocortical information and, in this case, inhibits the medial temporal lobe which leads to more efficient cortical learning. Indeed, congruent items were associated with lower power as compared to incongruent items and this effect was reduced in older adults (Fig. 7) who, supposedly, have structurally and functionally impaired prefrontal cortices^9^. Clearly, these neural oscillations may not necessarily originate within the prefrontal cortex; therefore, future studies may address this more directly, for instance, by using combined EEG/fMRI, which would also allow to quantify structural changes in the prefrontal cortex and medial temporal lobe.

We would like to point out that, comparable to our findings, previous memory studies did not find differential effects for the theta and (low) alpha band questioning their functional dissociation during long-term memory processes^48^. While this could also represent a spill-over between neighboring frequency bands (due to the inherent limitations of TF measurements), future studies will need to further examine this open question. Along the same lines, the behavioral effects only paralleled the pattern of neural oscillations but a correlation was observed with ERPs (Fig. 5). Therefore, the supposed relationship between behavior and neural oscillations is indirect.

The ERP effects found here are comparable with previous age-related differences of the N400^34^, and studies showing that the FN400 indexes memory-related processes^25,31^. However, they are also compatible with the notion of a link to conceptual priming processes^26^ that facilitate successful encoding. In particular, the age-related ERP differences found here within the N400 time window were greater for congruent words than for incongruent words, suggesting age-related differences in processing of congruent or expected words and not incongruent stimuli. In addition, the N400 ERP difference between congruent and incongruent words was greater for remembered as opposed to forgotten words, suggesting a critical role in encoding processes. Indeed, individual differences in mean N400 amplitude predicted the differential memory advantage for congruent compared to incongruent words. Interestingly, we found a memory by congruence interaction for low-beta power, as we did for the N400 component, further supporting a link between these two measures^82^. Furthermore, the congruence and age-related differences in theta and alpha power also mirror, albeit to a lesser extent, the results from the N400 analysis lending some support to the idea of theta and alpha power being linked with N400 related processes^83^.

Taken together, semantic congruence promotes long-term memory in both young and older adults, with only weak evidence of age-related impairments. At the neural level, the congruence effect could be linked to neural oscillations in the theta, alpha and beta range as well as ERPs, which have previously been associated with semantic processing.

## Supplementary information

Supplementary Information.
