# Supplementary material for: Neural oscillations and event-related potentials reveal how semantic congruence drives long-term memory in both young and older humans
Source: Sci Rep. 2020 Jun 4;10:9116. doi: 10.1038/s41598-020-65872-7 (PMC7272459; doi:10.1038/s41598-020-65872-7)
Supplement: Supplementary file 1 — Supplementary Information. [file 41598_2020_65872_MOESM1_ESM.docx]

**Neural oscillations and event-related potentials reveal how semantic congruence drives long-term memory in both young and older humans**

*Pau A. Packard^1,^ Tineke K. Steiger^1^, Lluís Fuentemilla^2^, *Nico Bunzeck^1^

(1) Institute of Psychology I, University of Lübeck, 23562 Lübeck, Germany

(2) Cognition and Brain Plasticity Group, Bellvitge Biomedical Research Institute (IDIBELL), Hospitalet de Llobregat, Spain; Department of Cognition, Development and Educational Psychology, University of Barcelona, Barcelona, Spain; Institute of Neurosciences, University of Barcelona, Barcelona, Spain

**Corresponding authors:*

Pau Packard and Nico Bunzeck

Institute of Psychology I, University of Luebeck

Ratzeburger Allee 160, 23562 Luebeck, Germany

Phone: +49-(0) 451 3101 3603

Email: pau.packard@gmail.com or nico.bunzeck@uni-luebeck.de

## Supplementary Results

## **Behavioral findings**

## Additional analyses excluding older subjects with MOCA scores under 26

### Experiment 1

#### Main effects of congruence, and age, for high-confidence corrected hit rate

The proportions of high-confidence ‘Sure’ responses during the recognition phase were analyzed. A 2x2 ANOVA with the factors congruence and age revealed a significant main effect of congruence (F_(1,49)_ = 425.07, p < 0.001, η_p_^2^ = 0.90), driven by higher CHR for congruent words, than for incongruent words. There was also a significant main effect of age (F_(1,49)_ = 5.06, p = 0.029, η_p_^2^ = 0.09), with higher CHR for younger, than for older participants. The congruence by age interaction was only trendlevel significant (F_(1,49)_ = 3.76, p = 0.058, η_p_^2^ = 0.07), which might be due to the slightly lower number of subjects.

### Experiment 2

#### Main effect of congruence for high-confidence CHR

In Experiment 2, the proportions of high-confidence ‘Sure’ responses during the recognition phase were analyzed, again, in a 2x2 ANOVA. This analysis only showed a significant main effect for congruence (*F*_(1,37)_ = 269.33, *p* < 0.001, η_p_^2^ = 0.88). In contrast to the behavioral experiment (Experiment 1), there was no main effect of age (*F*_(1,37)_ = 0.06, *p* = 0.810, η_p_^2^ = 0.02). The congruence by age interaction effect was not significant (*F*_(1,37)_ < 0.01, *p* = 0.931, η_p_^2^ < 0.01).

### Analysis of both experiments

#### Main effect of congruence for high-confidence CHR

A separate 2x2x2 ANOVA on high-confidence ‘Sure’ responses during the recognition phase, with congruence, age group, and experimental group as factors, including all the participants from both experiments, revealed a significant main effect of congruence (*F*_(1,86)_ = 677.90, *p* < 0.001, η_p_^2^ = 0.89), a non-significant effect of age (*F*_(1,86)_ = 1.93, *p* = 0.169, η_p_^2^ = 0.022); and a marginal effect of experiment (*F*_(1,86)_ = 3.56, *p* = 0.062, η_p_^2^ = 0.040). The congruence by age interaction effect was not significant (*F*_(1,86)_ = 1.56, *p* = 0.214, η_p_^2^ = 0.02). Finally, there were no other interactions between experimental group and age or congruence (all *p* > 0.080), which again, might be due to the lower number of subjects as compared to the analysis with a MoCA score of ≥ 22.
